# Second Trimester Amniocentesis Is Not a Risk Factor for Very Low Birth Weight and Extremely Low Birth Weight

**DOI:** 10.5402/2011/313206

**Published:** 2011-08-03

**Authors:** Vincenzo Mazza, Mariangela Pati, Emma Bertucci, Carlotta Cani, Silvia Latella, Giancarlo Gargano, Antonio Percesepe, Annibale Volpe

**Affiliations:** ^1^Prenatal Medicine Unit, Department of Obstetrics and Gynecology, University of Modena and Reggio Emilia, 41121 Modena, Italy; ^2^Department of Neonatal Intensive, University of Modena and Reggio Emilia, 41121 Modena, Italy; ^3^Medical Genetics, University of Modena and Reggio Emilia, 41121 Modena, Italy

## Abstract

*Objectives*. To assess the risk of very low birth weight (VLBW) and extremely low birth weight (ELBW) attributable to second trimester amniocentesis. *Methods*. Records of 4,877 consecutive amniocentesis, performed between 1997 and 2003, were analyzed. Only VLBW and ELBW in the study population (exposed) and in the control group (unexposed) were evaluated. Comparisons were made between the amniocentesis group versus nonexposed. Odds ratios (OR) and 95% confidence intervals (95% CI) were calculated for VLBW and ELBW classes. *Results*. In the study population, the VLBW were 35 (0.71%) and the ELBW were 20 (0.41%). In the control group, the VLBW were 220 (0.67%) and the ELBW were 112 (0.34%). The Odds ratios of the VLBW between the study and the control group did not show any statistical significant risk (OR = 1.07, 95% CI = 0.72–1.54). Also in ELBW odds ratios between study and control group were not statistically significant (OR = 1.20, 95% CI = 0.7–1.95). *Conclusions*. No effect of the second trimester amniocentesis was noted on VLBW and ELBW.

## 1. Introduction

Amniocentesis is an invasive prenatal diagnostic examination widely performed to screen fetal karyotypic abnormalities early in the second trimester of pregnancy. Despite the advent of the 1st trimester screening procedures for chromosomal aneuploidies, there is a growing number of procedures performed worldwide: in Italy, the number of procedures has raised from 50,527 in 1995 up to 101,750 in 2007 [1, 2]. 

Several studies have attempted to estimate the risks associated with amniocentesis conducted during the second trimester [3, 4]: most of them analyzed the risk of fetal loss related to the procedure, whereas only few studies were published concerning other possible complications, in particular the association between amniocentesis and preterm birth [5–7], especially the association between amniocentesis and ELBW (extremely low birth weight, less than 1000 gr) and VLBW (very low birth weight, less than 1500 gr), two categories of newborns representing approximately 1–1.5% of all live births, but contributing up to 40–60% of all neonatal and infant deaths and to 50% of neurological neonatal handicap [8–12].

The aim of the present study was to investigate whether amniocentesis performed during the 2nd trimester of pregnancy increase the risk of VLBW and ELBW.

## 2. Material and Methods

A total of 5043 women with singleton pregnancies underwent amniocentesis between 1997 and 2003 by a single operator; all amniocentesis were performed under ultrasound guidance in fetuses with a biparietal diameter (BPD) between 30 and 39 mm, as previously described [13].

Of these, 50 women (1% of the total) were lost to follow up and were excluded. Fetal karyotypic abnormalities were observed in 95 cases (1.9%), and 43 of them decided to terminate the pregnancy. In addition, 33 pregnancies were interrupted due to a major malformation. Moreover, also fetal losses were excluded from the present study: total fetal loss was 40 (0.81% of the total): 37 (0.76%) cases occurred before the 28th gestational week and 3 (0.06) cases after the 28th week of pregnancy, as previously published [13].

The clinical charts of the delivery in a total of 4,877 Caucasian women exposed to amniocentesis were reviewed, and the birth weight was recorded.

The pregnancy outcome was determined by clinical chart (80% cases) or by telephone interviews to the women (20% cases). For all the newborn with prematurity, the clinical chart was examined. 

The control group (unexposed to amniocentesis) was obtained evaluating VLBW and ELBW from 37,642 consecutive caucasian women who delivered in the same period in our district. The clinical charts were controlled to exclude women undergoing amniocentesis elsewhere.

Comparisons were made between the amniocentesis group versus nonexposed. Infant outcomes were given for the population of women giving birth to live infants.

Odds ratios (OR) and 95% confidence intervals (95% CI) were calculated for VLBW and ELBW classes. The comparison between our observed cases and the expected was expressed by the observed/expected rate (*O*/*E* rate).

## 3. Results

Out of 4,877 women undergoing amniocentesis, total VLBW were 35 (0.71%). Eleven of them were under the 10th centile and six of them under the 5° centile. ELBW were 20 (0.41%). Six of them were under the 10° percentile and three of them under the 5th centile. In the control group, the VLBW were 220 (0.67%) and the ELBW were 112 (0.34%). The Odds ratios of the VLBW between the study and the control group did not show any statistical significant risk (OR = 1.07, 95% CI = 0.72–1.54). Also in ELBW the odds ratios between the study and control groups were not statistically significant (OR = 1.20, 95% CI = 0.7–1.95) (Table 1).

## 4. Discussion

The present study is the first to analyze the risk of having ELBW and VLBW newborns after amniocentesis performed in second trimester and shows no statistically significant increase in these two categories of preterm newborns.

The importance of this findings lies in the severity of the VLBW newborns contributing to more than 50% of infant mortality and to 50% of neurological handicap [9–12], with a growing evidence that a significant number of these children have learning disability, attentional problems such as attention deficit hyperactivity disorder (ADHD), and minor motor problems [9].

Our findings are in agreement with those reported in a cohort study published by Tongsong et al. [7], in which the authors did not find statistically significant differences in fetal loss rate and premature delivery; Tabor et al. [6], in their randomized, controlled study, concluded that amniocentesis is not related to preterm delivery as well. Moreover, the most recent cohort study, based on data collected between 1991 and 1996 on 71,586 single births, showed that there is not an increased risk for limb reduction defects, fetal and infant mortality, prematurity, fetal distress, and low birth weight [14].

On the contrary, a positive association between preterm delivery and genetic amniocentesis has been described in the EUROPOP study Group (2003), a case-control study made by 10 European countries in which, however, a possible bias could be represented by the additional burden of complications contributed by four out of the 10 centers, performing amniocentesis between the 11th–15th week, when an increased risk of complications is reported [15, 16].

In conclusion, the absence of increased risk of preterm delivery associated with genetic amniocenteses performed in the second trimester of pregnancy makes unnecessary the information about this complication during the preamniocentesis counseling.

## Figures and Tables

**Table 1:** Number of ELBW and VLBW in study population and in control group. The odds ratio values between the two classes of weight in the control group and in the study group did not show any statistical significant risk.

Weight	Cases		Controls		*O*/*E*	95% CI
	*N*	*%*	*N*	*%*		
ELBW	20	0.41	112	0.34	1.20	0.72–1.54
VLBW	35	0.71	220	0.67	1.07	0.70–1.95
Tot.	4877		32987			
